# Individual, Contextual, and Age-Related Acoustic Variation in Simakobu (*Simias concolor*) Loud Calls

**DOI:** 10.1371/journal.pone.0083131

**Published:** 2013-12-23

**Authors:** Wendy M. Erb, J. Keith Hodges, Kurt Hammerschmidt

**Affiliations:** 1 Department of Anthropology, Rutgers University, New Brunswick, New Jersey, United States of America; 2 Reproductive Biology Unit, German Primate Center, Göttingen, Germany; 3 Cognitive Ethology Laboratory, German Primate Center, Göttingen, Germany; Université de Strasbourg, France

## Abstract

Primate loud calls have the potential to encode information about the identity, arousal, age, or physical condition of the caller, even at long distances. In this study, we conducted an analysis of the acoustic features of the loud calls produced by a species of Asian colobine monkey (simakobu, *Simias concolor*). Adult male simakobu produce loud calls spontaneously and in response to loud sounds and other loud calls, which are audible more than 500 m. Individual differences in calling rates and durations exist, but it is unknown what these differences signal and which other acoustic features vary among individuals. We aimed to describe the structure and usage of calls and to examine acoustic features that vary within and among individuals. We determined the context of 318 loud calls and analyzed 170 loud calls recorded from 10 adult males at an undisturbed site, Pungut, Siberut Island, Indonesia. Most calls (53%) followed the loud call of another male, 31% were spontaneous, and the remaining 16% followed a loud environmental disturbance. The fundamental frequency (F0) decreased while inter-unit intervals (IUI) increased over the course of loud call bouts, possibly indicating caller fatigue. Discriminant function analysis indicated that calls were not well discriminated by context, but spontaneous calls had higher peak frequencies, suggesting a higher level of arousal. Individual calls were distinct and individuals were mainly discriminated by IUI, call duration, and F0. Loud calls of older males had shorter IUI and lower F0, while middle-aged males had the highest peak frequencies. Overall, we found that calls were individually distinct and may provide information about the age, stamina, and arousal of the calling male, and could thus be a way for males and females to assess competitors and mates from long distances.

## Introduction

Vocalizations are commonly used in long-distance animal communication, as sounds have the potential to carry complex information even at long range [1]. This is especially true in tropical forests where visibility is severely limited, and vocal signaling may be the primary means of gaining information from conspecifics [2]. Long-distance vocalizations (known as loud or long calls) are widespread among non-human primates (reviewed in [3]–[4]). These calls are loud, conspicuous vocalizations that carry over long distances and typically show specializations for transmission, including rapid rise times, broad frequency bandwidths, and relatively low frequencies [2], [5].

Auditory signals may encode information about attributes of the sender, such as identity, sex, body size, age, rank, physical condition, and fatigue [6]–[13]. They may also contain information about the context of the call, such as the presence of food, predators, or social conflict [14]–[18], as well as the arousal or motivation of the caller [19]–[21]. This information can be found in the spectral (i.e., frequency dimension) as well as temporal attributes of calls.

Spectral features are determined by the vocal folds as well as the filter function of the vocal tract (reviewed in [10], [22]). For example, the fundamental frequency (F0) is the primary determinant of perceived pitch and is controlled by vocal fold size and tension, with longer, thicker, and more relaxed folds producing lower-pitched sounds [22]. This parameter has been shown to vary with the caller's identity (e.g., African elephants, *Loxodonta africana* [23], Iberian wolves, *Canis lupus* [24]), body size (e.g., Japanese macaques, *Macaca fuscata* [25], hamadryas baboons, *Papio hamadryas* [26], chacma baboons, *Papio ursinus* [27]), rank (e.g., chacma baboons [8], chimpanzees, *Pan troglodytes* [28], fallow deer, *Dama dama* [29]), age (hamadryas baboons [8], [16], arousal (e.g., chacma baboons [30], humans [31], spotted hyenas, *Crocuta crocuta* [11], African elephants [32]), and even reproductive success (e.g., red deer [33]). Listeners might use some or all of this acoustic information to identify infanticidal males (e.g., lions, *Panthera leo* [34]), differentiate neighbors from intruders (e.g., Thomas langurs [3]), recognize the presence of predators (e.g., vervet monkeys, *Chlorocebus aethiops* [14]), or identify a strong competitor or high-quality mate (e.g., red deer, *Cervus elaphus* [35]–[36]).

Temporal features of calls vary with the lung capacity of the caller as well as the control and timing of the emptying speed of air. For instance, call duration is related to lung capacity, and the size of the lungs, in turn, is closely related to body size [37]. Call duration has been shown to signal the stamina of the caller if calls are energetically difficult to produce (cf. [38]). Indeed, call duration has been linked to the age (e.g., chacma baboons [8]), rank (e.g., guerezas, *Colobus guereza* [9]), physical condition (e.g., fallow deer [13]), and fighting ability (e.g., Thomas langurs [39]) of the caller.

In the current study, we present an analysis of the acoustic features of the loud calls produced by a species of Asian colobine, the simakobu (*Simias concolor*). Loud calls are common among Asian colobine males and are thought to play a role in mediating group spacing and preventing encounters and fights between groups [40]. Most of what is known about Asian colobine loud calls comes from studies of one species, the Thomas langur. Behavioral observations, acoustic analyses, and playback studies have revealed that calls produced by different individuals and in different contexts are acoustically distinct [17], [41]. Individual callers are discriminated by both temporal and spectral variables, with measures of the fundamental frequency showing the greatest differences among individuals. Call contexts, on the other hand, are best discriminated by temporal variables, including duration and inter-unit intervals. Males of different ages also produce calls of varying durations and rates [39]. The loud calls of other Asian colobines have not been well studied (but see [42]), and it is unknown whether the patterns found in Thomas langurs can be generalized to other species.

Simakobu loud calls are produced by males only and are audible from distances exceeding 500 m, even in their dense rainforest habitat ([43], WM Erb, pers. obs.). They are reported to occur in a number of contexts: 1) spontaneously, 2) in response to the loud calls of other males, often as part of a chorus, 3) in response to loud environmental disturbances, such as tree falls or thunder, and 4) during intergroup encounters [43]. Males frequently produce loud calls at dawn, and these dawn calls usually occur as a chorus of two or more callers [44]–[45]. Previous research suggests that these vocalizations may function, in part, as honest advertisements of male energy status in this species [45]. Adult males in one-male groups exhibit exclusive use and aggressive defense of areas, and since groups meet and interact infrequently, loud calls are likely used by listeners to assess callers from long distances [45].

While previous research indicates that there are significant individual differences in simakobu calling rates and call durations [45], it is unknown what these differences may signal and which other acoustic features vary among individuals. Thus, the objective of the present study is to determine which acoustic features vary within and among callers and whether these differences might signal aspects of male fighting ability (e.g., age, stamina, arousal). Because these vocalizations are also made in a variety of contexts, we examine whether loud calls made in different contexts are acoustically distinct. We first provide a general description of the acoustic properties of loud calls and the contexts in which they are produced. We then examine the sources of variation in these calls. We begin with an investigation of short-term variation (i.e., within a single call bout), followed by analyses of variation across contexts, individuals, and age classes to identify the acoustic features that contribute most to their discrimination. If loud calls are honest signals of male competitive ability, we expect acoustic features to vary among individuals and males of different ages.

We focus on four acoustic parameters that have been shown to exhibit variation among callers and contexts: call duration, inter-unit interval, fundamental frequency, and peak frequency. Based on sound production mechanisms as well as the results of previous research, we make several predictions. Over the course of a loud call, we expect that, as the lungs deflate and/or the caller becomes fatigued, there will be a decrease in the fundamental frequency and an increase in the inter-unit interval (cf. [8], [13], [22]). We expect that calls produced in different contexts will be distinguished by the level of arousal of the caller, with more stressful or aversive states reflected in decreased inter-unit interval, increased duration, and increased frequency characteristics (cf. [15], [21], [30], [46]–[47]). Finally, we expect calls to convey differences in the relative ages of callers and predict that prime-aged males will have longer calls with shorter inter-unit intervals and lower fundamental frequencies (cf. [8]).

## Methods

### Study site and subjects

Research was carried out at the Pungut study site in the Peleonan Forest in northern Siberut, Indonesia (0°56′–1°03′S, 98°48′–98°51′E), a 10.7-km^2^ area of hilly (altitude: ca. 25–190 m) primary evergreen rainforest. This area is managed by the Siberut Conservation Programme and is protected from hunting and logging through agreements with Indonesian officials and the local community. The climate is equatorial with mean monthly temperatures ranging from 21.5 and 31.7°C, and mean annual precipitation of 3,601 mm [48]. In addition to simakobu, three other primate species inhabit the study area: Kloss' gibbon (*Hylobates klossii*), Mentawai langur (*Presbytis potenziani*), and Siberut macaque (*Macaca siberu*). With the exception of humans, mammalian predators do not occur on the Mentawai Islands, and potential predators are limited to serpent eagles (*Spilornis cheela sipora*) and reticulated pythons (*Python reticulatus*) [49].

Simakobu at this site reside in one-male groups (OMGs) with 3.0 females (range = 2–5) and 7.9 individuals on average, as well as in all-male groups (AMGs) averaging 4.5 individuals [50]. Female dispersal is common, and group home ranges are small (<10 ha), exhibiting little overlap between adjacent groups [45], [50]. Study subjects were 10 adult males residing in OMGs (*N* = 9) and AMGs (*N* = 1). Four of the study groups were habituated to observers in 2006/2007, and all individuals were identified (details in [50]). Two of these groups (OMG-H = 130 follow days; and AMG-D = 104 follow days) were followed beginning in February 2007, and most group members were habituated by May 2007. Two other habituated groups (OMG-A = 19 follow days; and OMG-E = 21 follow days) were observed at the end of the study (June–December 2008). The remaining six OMGs (C = 5 follow days; F = 16 follow days; J = 19 follow days; P = 9 follow days; S = 113 follow days; and Z = 25 follow days) were unhabituated neighboring groups. They were contacted during July–August 2005 and February 2007–December 2008 at irregular intervals.

### Data collection

Data were collected during the 25-month study period (July–August 2005 = 40 days; and February 2007–December 2008 = 173 days) by WME and research assistants (see Acknowledgments). Groups were typically followed to sleeping trees in the evenings and relocated the following morning. Unhabituated groups were identified by their location within the study area, as well as distinctive features of the adult males and other easily recognized group members. Individuals were identified by the shape and hair patterns of the tail, by the size and shape of the crest, and by the patterns and coloration of the facial hair.

During contact with groups, we employed all-occurrence sampling of loud calls [51]. Whenever a loud call was heard, we recorded the following: time, stimulus (loud call, tree fall, thunder, airplane), and the caller(s)' identity and location (GPS point). Recordings were made *ad libitum* by WME with a Marantz PMD-660 Solid State recorder (48 kHz sampling frequency, 16 bit; Marantz, Japan) and Sennheiser directional microphone (K6 power module, ME66 recording head; Sennheiser, Wedemark, Germany). Recording distances between observer and caller ranged from 11–200 m, with an average distance of 32.6 m (23.6 m for habituated callers, 52.6 m for unhabituated callers). Maximum amplitudes (dB) of loud calls were also occasionally measured by WME with a Sinometer JTS1357 digital sound level meter (Sinometer, Shenzhen, China).

### Acoustic analysis

We accumulated 186 loud call recordings of sufficient quality for acoustic analysis during the study period. Prior to analysis, we visually inspected calls at a sample frequency of 11,025 Hz using Cool Edit 2000 (Syntrillium, Phoenix, AZ), and selected recordings that were not cut off or disturbed by background noise (e.g., birds, insects, other loud calls). Prior to the spectral analysis, we used a FFT filter (−30 dB) in Cool Edit to remove low-frequency (<100 Hz) and high-frequency (>5000 Hz) noise from recordings. We then used Avisoft SASLab Pro (Avisoft Bioacoustics, Berlin, Germany) to create spectrograms (FFT length: 1024 points, window: Hamming, frame size: 100%, overlap: 93.75%).

Spectrograms were visually inspected to determine the start and end point for each call. Due to a high degree of variability in call units near the end of loud calls (e.g., sometimes males continued vocalizing for several seconds or minutes following a loud call), it was occasionally difficult to determine the end point of a particular call. To address this issue, we measured the duration of the interval between successive call units within a sample of 38 recordings made during the pilot study in 2005 and generated a histogram (class width = 0.25 s) of these durations. From the histogram, we were able to identify a change point in the distribution at 2.0 seconds. We confirmed this result using Change Point Analyzer 2.3 (Taylor Enterprise, Inc.), which estimated class 8 (2 s) as the most likely time of change, and used this to define the end of loud calls. In other words, once the duration between two successive call units exceeded 2.0 seconds, this was considered to be the end of the call. Once the start and end point for each call was determined, we measured its duration and counted the number of call units it contained.

**Figure 1:**
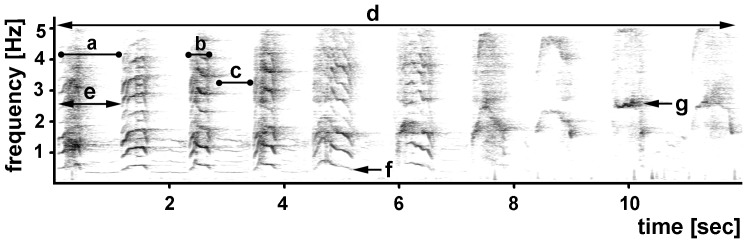
Sample spectrogram of a simakobu loud call indicating the parameters measured in the acoustic analysis: (a) call unit, (b) huh, (c) hoo, (d) duration, (e) inter-unit interval, (f) fundamental frequency, and (g) peak frequency.

Simakobu loud calls are produced as a series of one- or two-syllable call units, each consisting of a loud noisy bark syllable (“huh”), typically accompanied by a quieter gasp syllable (“hoo”), particularly in the call units at the beginning of loud calls (Fig. 1). For the spectral analysis, each syllable of each call unit was saved as a separate file before generating the spectrograms in Avisoft (details above). The resultant spectrograms were then imported into LMA 2007, a custom software program. We used the interactive harmonic cursor tool to extract the acoustic parameters from the calls. This tool projects multiple lines with integer intervals of the cursor. This enables the observer to visually determine whether a given spectrogram has a periodic (harmonic) characteristic, and to identify the lowest harmonic (F0). The F0 value is then measured by the program, with an algorithm that searches for the frequency with the highest amplitude within the range of the cursor. In total, we examined six temporal and spectral acoustic parameters: call duration (i.e., duration from the start of the first unit until end of last unit), inter-unit intervals (i.e., duration of the interval between successive call units), as well as the fundamental frequency (i.e., lowest frequency of a harmonic series) and peak frequency (i.e., frequency with the highest amplitude) of the huh and hoo syllables.

### Data analysis

In some cases, the spectral parameters could not be measured for both syllables in a call unit. In order to minimize biases due to uneven sampling among calls, we randomly chose five huh and five hoo syllables from each loud call. In cases where fewer than five were available, data from all call units were used (10.2% of calls). To confirm the classification of huh and hoo syllables, we used a matched-samples t-test to analyze differences in the frequency characteristics of these two syllable types. A few calls exhibiting outliers in acoustic measures were replaced with more typical calls (1.1% of calls). Prior to analysis, we screened the data to look for any effects of recording distance on acoustic parameters (cf. [52]). We found a negative trend (*P*<0.10) for the effect of distance on peak frequency when all recordings were included, but this relationship disappeared when we removed those calls recorded at distances exceeding 75 m. Thus, we excluded these calls (*N* = 16) recorded from longer distances.

To examine whether the acoustic properties of vocalizations changed over the course of a single loud call, we selected calls with data available for most call units and both syllable types (mean = 90%, range = 81–100% complete; *N* = 22 loud calls, 4 males). For this analysis, we calculated the relative position of each call unit, ranging from 0 to 1, wherein 1 indicated the last unit. For example, in a call with 10 units, the first unit position = 0.1, the second = 0.2, and so on. We then analyzed the relationship between call unit position and acoustic properties (fundamental frequency, peak frequency, and inter-unit interval).

Calls were assigned to one of three contexts: noise, social, or spontaneous. Calls produced within five minutes of a loud disturbance, including thunderclaps, tree falls, airplanes, or branch breaks were assigned to the “noise” context. “Social” calls were defined as those produced within five minutes of another loud call that was not preceded by a loud noise. Calls produced in a chorus following a loud noise, however, were assigned to “noise”. Calls that occurred without any apparent auditory stimulus were classified as “spontaneous”.

To assess age-related variation, we compared calls across males residing in seven groups. Although their exact ages were not known, they could be ranked relative to each other. Three males (JK, SM, and ZS) were classified as “older” because they were fully grown (head-body length) at the beginning of the study in 2005, and resided in mixed-sex groups for the duration of the study. Males in the “middle” class (AL and HL) reached adult head-body length and established mixed-sex groups in early 2007. “Younger” males (DG and EL) were not fully grown and resided in all-male bands in 2007. In 2008, they were fully grown and began to produce loud calls. Thus, the average difference between adjacent age classes is estimated to exceed 1.5 years.

We performed discriminant function analyses (DFA) to test whether calls could be reliably classified according to context, caller identity, and age class. To prevent results from being over-represented by males for whom more calls were available and to minimize the confounding effects of individual differences, we limited the sample for the contextual analysis to males who contributed at least one call to each context and selected up to 10 calls per male. For the DFA of individual differences and age classes, we selected up to 15 calls per male. When testing for individual differences, we included only those individuals who contributed at least six calls, and selected calls produced in the “social” and “noise” contexts only (except one “spontaneous” call from male SM). Although we acknowledge that a higher number of calls per individual would have been better, we were limited by the overall sample size available. We subsequently used a permuted discriminant function analysis (pDFA), using an R algorithm written by Roger Mundry, to assess whether our classification results could have been due to chance [53]. To test for age differences, we used a nested design controlling for individual influence (six subjects). To test for contextual and individual differences we used a crossed design. Because the crossed design requires complete data sets, we reduced our data to four subjects for which we had calls in all three contexts.

For each DFA, variables were entered using the direct method and call classification was cross-validated using the leave-one-out procedure. We checked for outliers and tested the assumption of homogeneity of covariance by plotting the first two functions to check for extreme values and confirm that the spread of points was similar among groups (cf. [54]). Following DFA, we conducted univariate general linear mixed models (LMM) using the Hochberg procedure to correct for multiple testing [55]. We recognize that ideally one would use one set of data for these sets of analyses; however, small sample sizes limited our ability to do this.

To analyze changes in acoustic properties within a loud call, we conducted univariate LMMs with call unit position as a fixed factor and the recording ID as a random factor. To analyze differences due to age and context, we entered individual ID as a random factor and the mean position of the call unit as a covariate. We further tested for interaction effects between our predictor variable and ID, as well as between age and context. When the covariate and interaction effects were not significant, we removed them from the models and reported results without these effects. Before conducting DFA, we checked for univariate and multivariate outliers following [56]. We identified outliers in LMM analyses as cases with studentized residual scores exceeding an absolute value of 3.0 (cf. [57]). We re-ran those analyses without outliers and compared results. When the removal of outliers did not affect the patterns and significance of our results, we retained these cases and report test results with them. Statistical tests were performed using SPSS 19 (for DFA), R (for pDFA), and Statistica 9 (for LMM) with alpha set at *P*<0.05.

### Ethics statement

Research permits were issued by the Indonesian Institute of Sciences (LIPI Research Permit #0604/SU/KS2007) in accordance with the legal requirements of conducting research in Indonesia. All research methods were approved by the Institutional Animal Care and Use Committee at Stony Brook University (Project ID: 2005–2008–1451).

## Results

### Contexts and acoustic properties of loud calls

Our description of the acoustic properties of calls was based on 170 full loud call recordings. For some calls, acoustic data for one or more huh or hoo syllables could not be measured (sample sizes: *N* = 162 calls for huh and *N* = 160 calls for hoo descriptions: Table 1). On average, calls were 15.5 s in duration and consisted of 15.9 call units (range = 5–31), with an average inter-unit interval of 1.0 s (Table 1). Huh syllables showed significantly higher frequency characteristics than hoo syllables. Fundamental frequency averaged 1310.4±167.2 SD Hz in huh syllables, and 867.0±143.2 SD Hz in hoo syllables (*t* = 30.53, df = 157, *P*<0.01). Similarly, peak frequency of huh syllables averaged 3798.4±803.3 Hz, while hoo syllables averaged 3307.0±666.4 Hz (*t* = 7.29, df = 157, *P*<0.01). We obtained sound pressure readings for 34 of these calls. Calls measured at 11–15 m (*N* = 3) were 76.7 dB on average (range = 73.5–79.5), while those measured at 20–30 m (*N* = 7) averaged 66.3 dB (range = 64.1–71.8). Even at distances exceeding 30 m, sound pressure levels were still high, ranging from 50.1–70.1 dB (*N* = 5).

**Table 1:** Descriptive statistics of acoustic parameters.

Variable	*N*	Mean	Min	Max	SD	CV
IUI [s]	170	1.00	0.69	1.48	0.165	16.485
Duration [s]	170	15.53	4.78	33.08	4.711	30.338
F0 (huh) [Hz]	162	1310.37	875.0	1905.8	167.197	12.759
Pf (huh) [Hz]	162	3798.39	2583.2	6466.0	803.267	21.148
F0 (hoo) [Hz]	160	866.97	461.0	1227.8	143.196	16.517
Pf (hoo) [Hz]	160	3306.99	1205.4	5714.0	666.421	20.152

Min = minimum value, Max = maximum value, SD = standard deviation, CV = coefficient of variation.

During the study period, we observed 318 loud calls while in close proximity (<50 m distance) to the caller. Of these, 167 (52.5%) followed the loud call of another male (“social” context), 99 (31.1%) were spontaneous, and 52 (16.4%) followed a loud disturbance (“noise” context: airplane: *N* = 19, tree fall: *N* = 19, thunder: *N* = 4, branch break: *N* = 1). Although we observed one loud call during one of the more than 50 intergroup encounters we witnessed (i.e., two groups were <50 m apart), this call immediately followed a loud branch break, and did not appear to occur as part of an agonistic display or interaction between the males.

### Short-term changes in acoustic features

**Figure 2:**
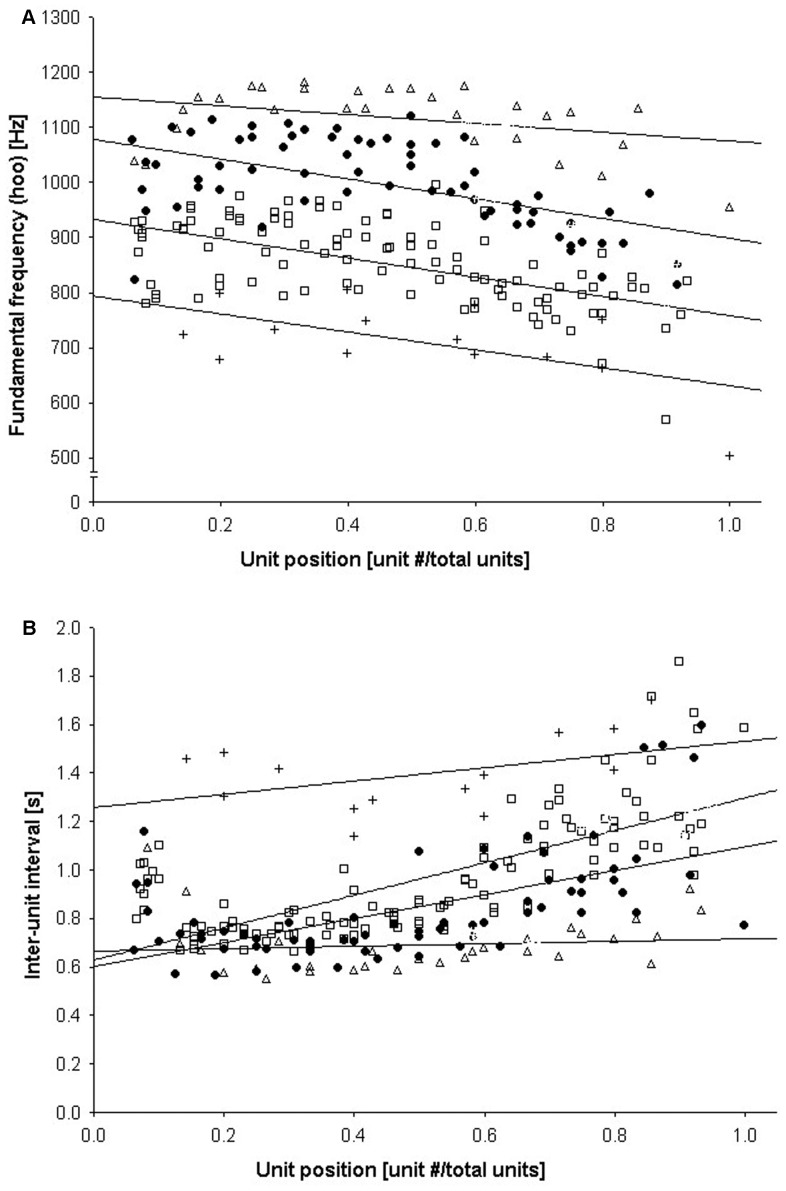
Changes in call characteristics as a function of unit position: (A) fundamental frequency of the hoo syllable and (B) inter-unit interval. Best-fit lines for individual males provided for demonstration purposes only.

In order to describe changes that occur during the progression of loud calls, we selected 22 calls with data available for most call units and both syllable types. Both the spectral and temporal properties of individual call units showed significant changes in relation to their position within loud calls, even after p-values were corrected for multiple testing (Table 2). For huh syllables, the fundamental frequency increased as the call progressed. For hoo syllables, on the other hand, fundamental frequency showed a decrease (Fig. 2). Peak frequency increased in both huh and hoo syllables across the call. Finally, the interval between successive call units showed an increase from the start to finish of the loud call (Fig. 2). These changes were greatest for inter-unit intervals and fundamental frequency of the hoo syllables, indicated by the large F-values for these variables and correspondingly high R^2^ values for the models.

**Table 2:** Variation in relation to call unit position within the loud call.

	Fixed effects					Random effects		Full model	
Variable	*F*	df	*P*	*β*	SE *β*	*F*	*P*	Mult *R* ^2^	*P*
IUI	127.421	1, 207	<0.001	0.502	0.044	8.178	<0.001	0.592	<0.001
F0 (huh)	70.155	1, 218	<0.001	0.445	0.053	3.462	<0.001	0.393	<0.001
Pf (huh)	26.448	1, 219	<0.001	0.278	0.054	5.023	<0.001	0.368	<0.001
F0 (hoo)	130.852	1, 188	<0.001	−0.294	0.036	58.060	<0.001	0.879	<0.001
Pf (hoo)	28.799	1, 190	<0.001	0.332	0.062	2.461	<0.001	0.291	<0.001

Results based on LMM with loud call recording as a random factor. *N* = 22 loud calls recorded from four males.

### Contextual differences

**Figure 3:**
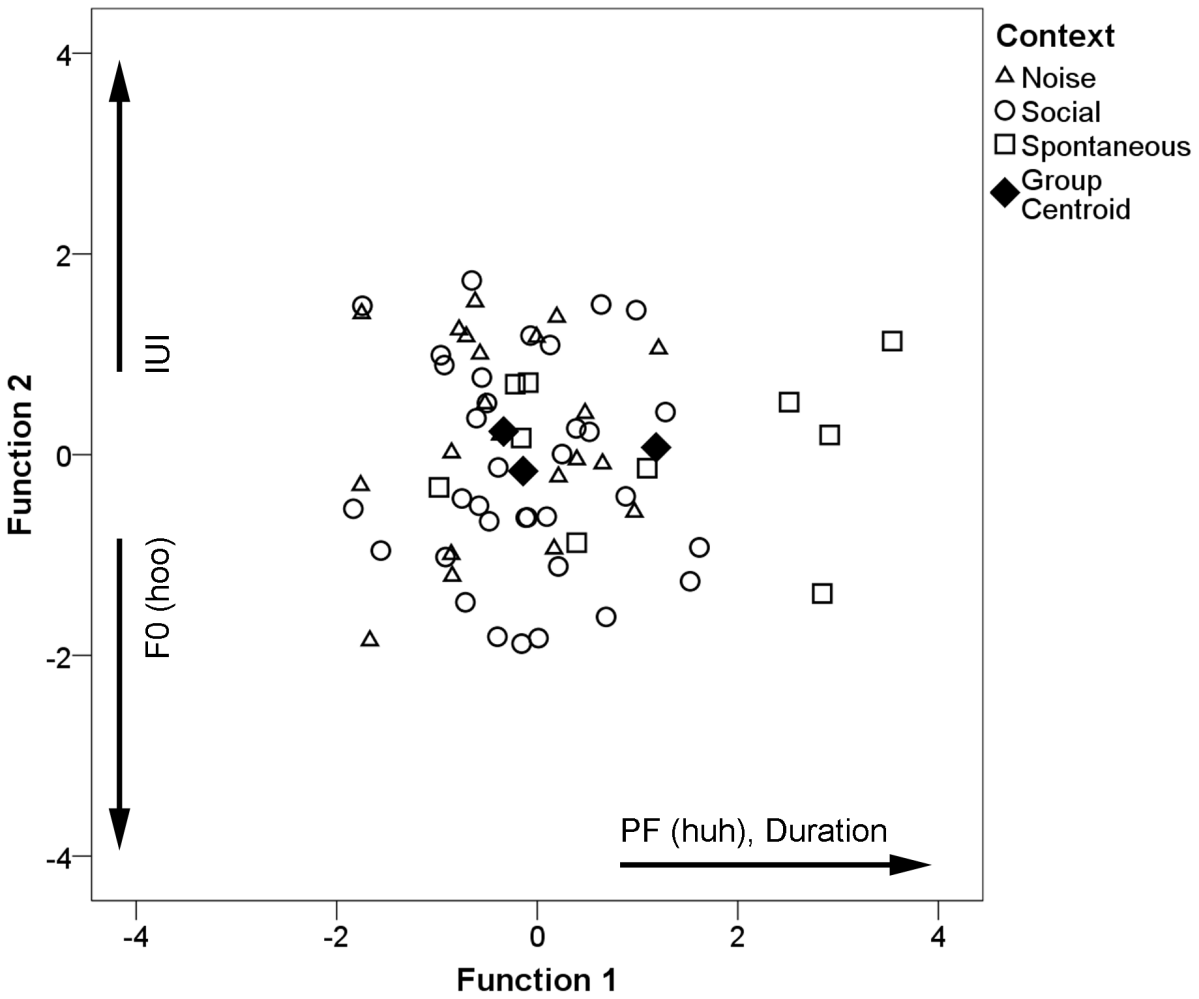
Discriminant scores for loud calls produced in different contexts for the first two canonical discriminant functions. The three contexts are denoted by different symbols. Variables showing loadings with an absolute value >0.4 for each function are indicated along the axes.

To evaluate the variation in loud calls across contexts, we conducted a discriminant function analysis of 65 calls recorded from four males (Table 3). The first function accounted for 89.6% of the variance. The average correct assignment was 58.5% of cases and the cross-validation procedure yielded an average correct assignment of 41.5%, indicating there was substantial overlap among contexts (Fig. 3). Calls were correctly assigned (cross-validation values in parentheses) to “noise” in 19.0% (4.8%), “social” in 88.2% (64.7%), and “spontaneous” in 40.0% (40.0%) of cases. Compared to their prior probabilities, calls produced in social and spontaneous contexts were correctly classified more than expected; however, the overall probability of obtaining these classification results was not different from chance (cross-validated pDFA: *N* = 65, *P* = 0.562). Spontaneous calls were discriminated from noise and social via the discriminant function scores for the first function, while the second function showed little separation among the three groups (Fig. 3).

**Table 3:** Number of calls for each male in each context.

			Context		
ID	Age	*N* *	Noise	Social	Spontaneous
AL^1^^, ^ 2	Middle	23	2	18	3
CH		1	0	1	0
DG	Younger	3	0	3	0
EL^1^^, ^ 2	Younger	25	8	15	2
FR		2	1	1	0
HL^1^^, ^ 2	Middle	66	15	46	4
JK^2^	Older	11	3	7	0
PC		2	1	1	0
SM^1^^, ^ 2	Older	6	1	4	1
ZS^2^	Older	8	1	6	0
Unk		23			
Σ		170	32	102	10

Unk = unidentified caller;

1 indicates calls selected for contextual analysis;

2 indicates calls selected for individual analysis;

* context could not be determined for some calls.

**Figure 4:**
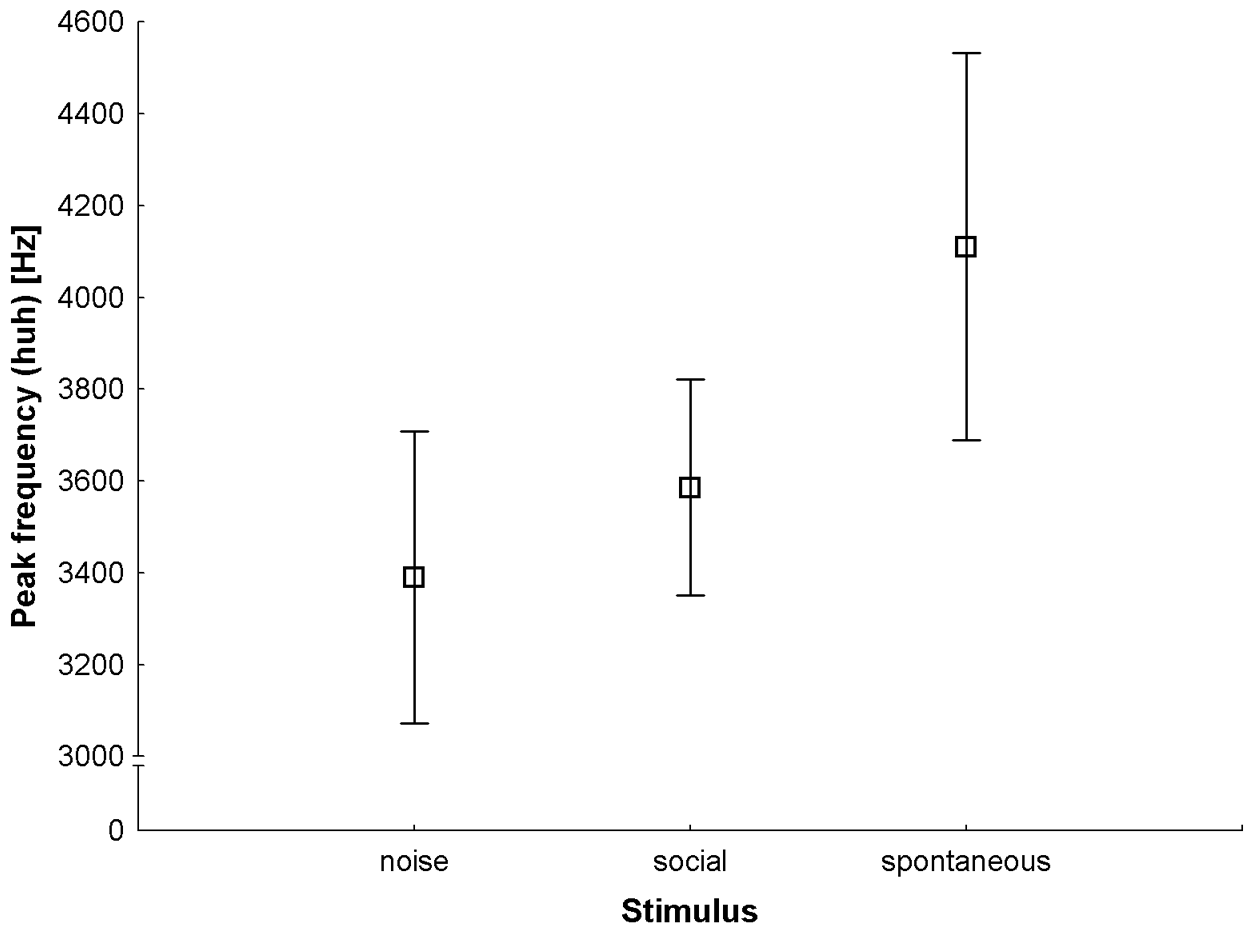
Mean ± 95% confidence interval for peak frequency of huh syllables across contexts.

The variables that contributed most to the discrimination of contexts, indicated by their large loadings on the first function (>0.45), were call duration and peak frequency of the huh syllable. The LMM revealed a significant effect of context on peak frequency (*F*_2, 59_ = 4.069, *P* = 0.022), though duration produced only a statistical trend (Table 4). There were no significant interactions between context and individual or age class, indicating that individuals showed similar patterns of loud call differences among contexts. Mean unit position also had no significant effect. In general, calls produced spontaneously were longer and showed higher peak frequencies than those made in other contexts (Fig. 4).

**Table 4:** Variation in relation to call context.

	Fixed effects			Random effects		Full model	
Variable	*F*	df	*P*	*F*	*P*	Mult. *R* ^2^	*P*
IUI	0.472	2, 59	0.626	20.256	<0.001	0.519	<0.001
Duration	2.709	2, 59	0.075	3.266	0.027	0.215	0.012
F0 (huh)	0.392	2, 59	0.678	2.723	0.052	0.134	0.121
Pf (huh)	4.069	2, 59	**0.022**	8.629	<0.001	0.350	<0.001
F0 (hoo)	0.115	2, 59	0.891	43.831	<0.001	0.693	<0.001
Pf (hoo)	0.661	2, 59	0.520	5.884	0.001	0.248	0.004

Results based on LMM with individual ID as a random factor. *N* = 65 loud calls recorded from 4 males.

Significant differences after Hochberg correction indicated in bold.

### Individual differences

**Figure 5:**
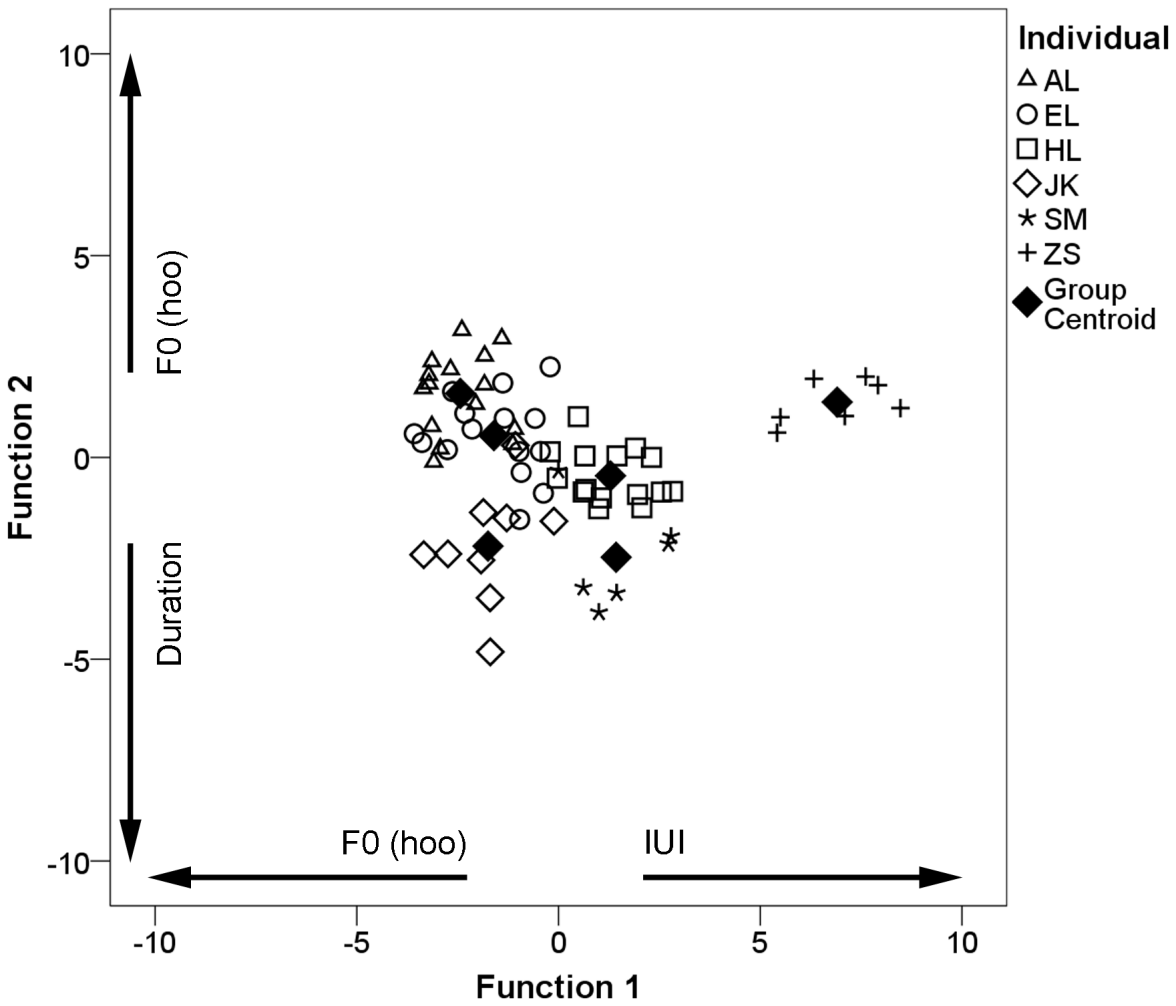
Discriminant scores for loud calls produced by different individuals for the first two canonical discriminant functions. The six males are denoted by different symbols. Variables showing loadings with an absolute value >0.4 for each function are indicated along the axes.

To evaluate the variation in loud calls by individual males, we conducted a discriminant function analysis of 67 calls recorded from six males. The first two functions accounted for 93.8% of the variance (Function 1: 74.2%, Function 2: 19.6%; Fig. 5). The assignment procedure of the discriminant function yielded an average correct assignment of 89.6% of cases, and the cross-validation procedure yielded an average correct assignment of 79.1%. Calls were correctly assigned to most males, with correct assignment scores ranging from 77.8–100% for each individual (60.0–100% cross-validated), and the probability of obtaining these classification results by chance was very low (cross-validated pDFA: *N* = 65, *P* = 0.01). Of the six variables entered into the analysis, three (inter-unit interval, duration, and fundamental frequency of the hoo syllable) contributed most to the discrimination of individuals, indicated by high loadings on the first two functions.

### Age differences

**Figure 6:**
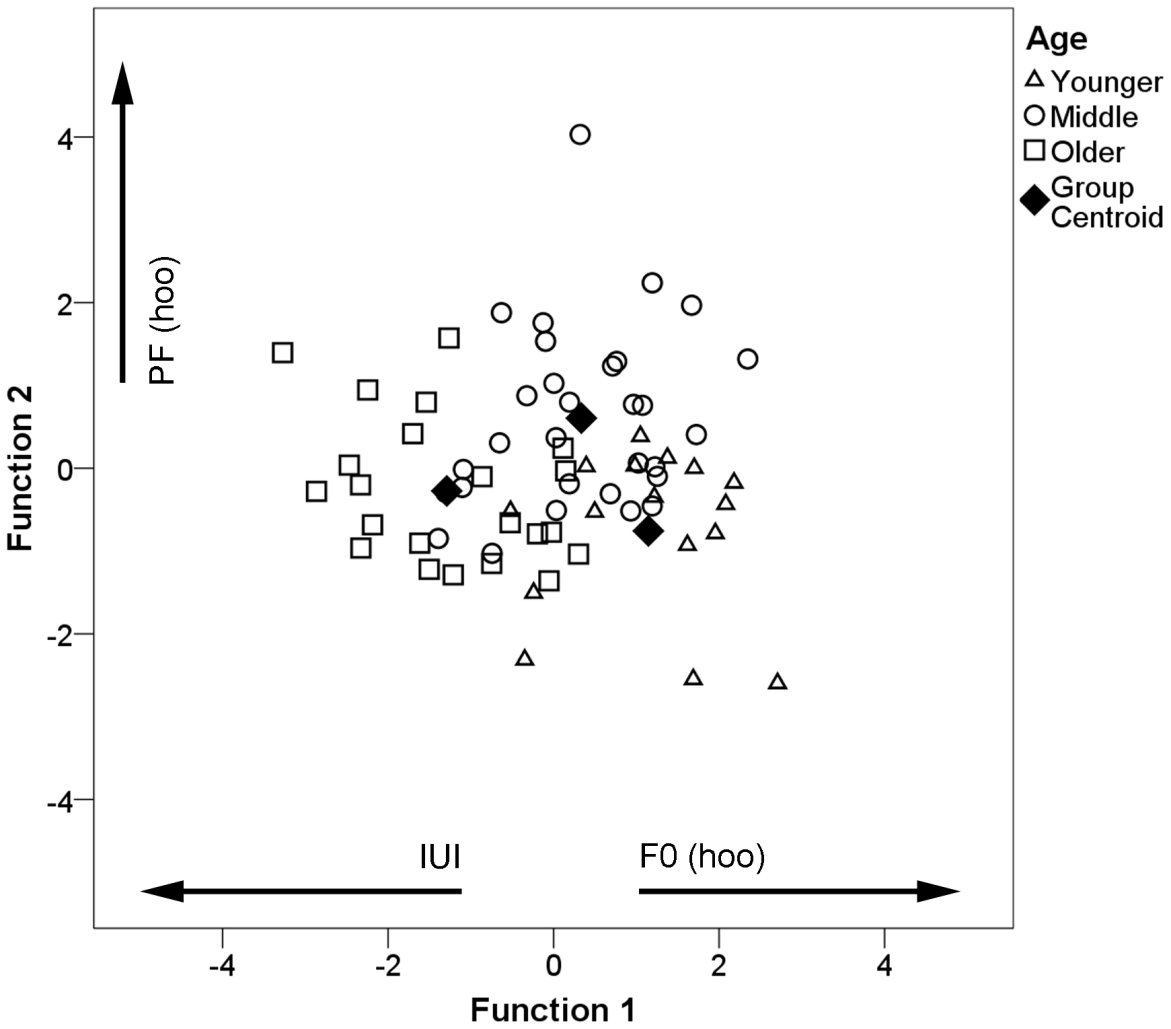
Discriminant scores for loud calls produced by males in different age classes for the first two canonical discriminant functions. The three age classes are denoted by different symbols. Variables showing loadings with an absolute value >0.4 for each function are indicated along the axes.

To evaluate the variation in loud calls among males of different relative ages, we conducted a discriminant function analysis of 68 calls from seven males (Table 3). The first function accounted for 73.7% of the variance (Fig. 6). The average correct assignment was 69.1%, with 60.3% of cross-validated cases correctly classified. Males in the younger age class were correctly classified in 50.0% (37.5% cross-validated), those in the middle age class 76.7% (66.7%), and males in the older age class 72.7% (68.2%) of cases; however, the overall probability of obtaining these classification results was not different from chance (cross-validated pDFA: *N* = 67, *P* = 0.751).

**Figure 7:**
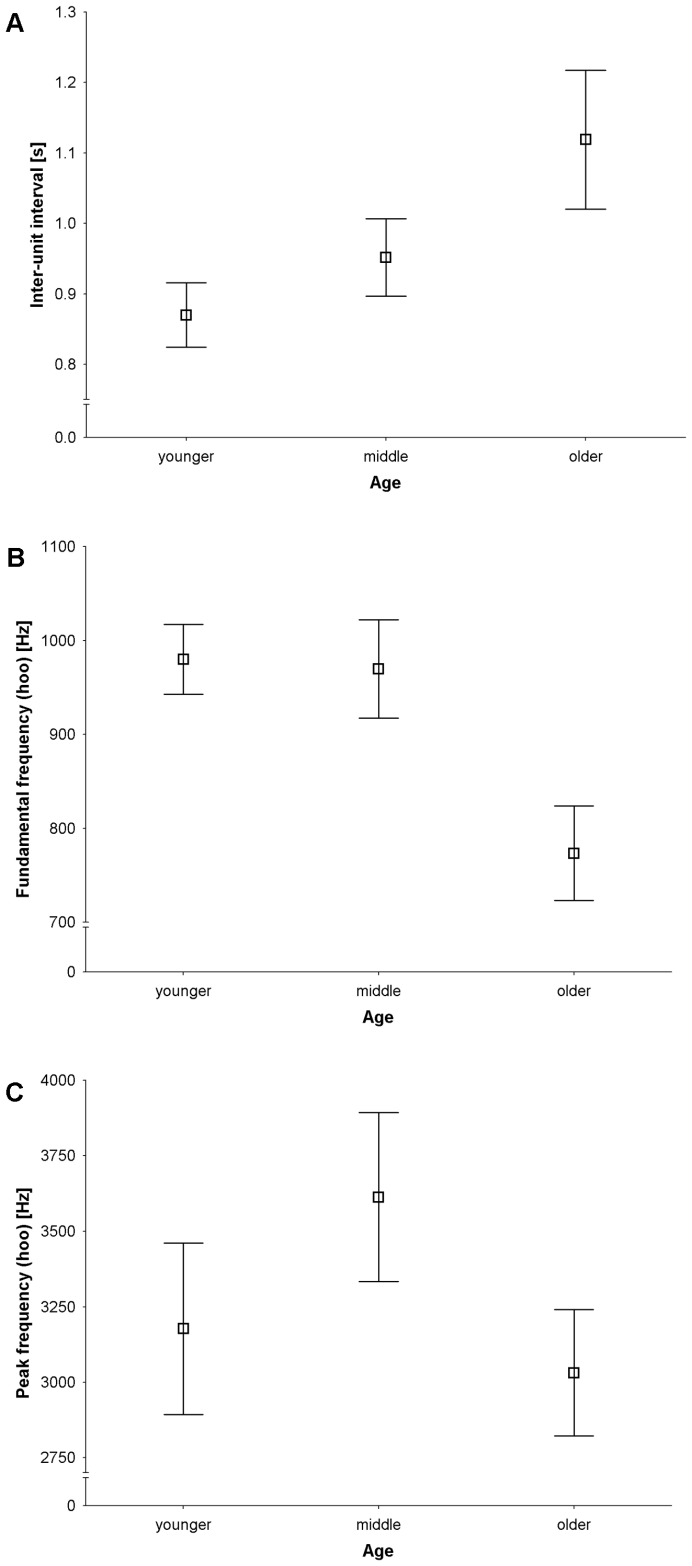
Mean ± 95% confidence interval of three parameters that varied significantly across age classes: (A) inter-unit interval, (B) fundamental frequency of hoo syllables, and (C) peak frequency of hoo syllables.

Inter-unit interval, peak frequency, and fundamental frequency of the hoo syllable contributed most to the discrimination of age classes. GLM analysis with Hochberg corrections revealed that inter-unit intervals increased with age (Table 5, Fig. 7a); while fundamental frequency of the hoo syllable decreased (Table 5, Fig. 7b). Peak frequency of the hoo syllable also differed significantly across age classes (Table 5), and appeared to be highest for males in the middle age class (Fig. 7c). Mean unit position had no significant effect in any of the models.

**Table 5:** Variation in relation to male age class.

	Fixed effects			Full model	
Variable	*F*	df	*P*	Mult. *R* ^2^	*P*
IUI	10.855	2, 65	**<0.001**	0.250	<0.001
Duration	1.851	2, 65	0.165	0.054	0.165
F0 (huh)	2.663	2, 65	0.077	0.076	0.077
Pf (huh)	2.520	2, 65	0.088	0.072	0.088
F0 (hoo)	14.356	2, 65	**<0.001**	0.306	<0.001
Pf (hoo)	7.510	2, 65	**0.001**	0.188	0.001

Results based on GLM. *N* = 68 loud calls recorded from 7 males.

Significant differences after Hochberg correction indicated in bold.

## Discussion

Like other species of Asian colobines, adult male simakobu produce loud calls that show specializations for long-distance transmission and distinct differences among individuals. Calls are high-amplitude and tonal, comprise redundant elements with modulating frequencies, and emphasize intermediate frequencies (1–4 kHz), features that promote long-distance propagation while reducing degradation during transmission [58]. Over the course of a single loud call, we found that call units showed significant changes as the call progressed, possibly indicating caller fatigue. Simakobu loud calls were produced in relatively limited contexts, which overall did not show distinct acoustic differences. Conversely, individuals were well discriminated by both spectral and temporal features of their calls, and we found significant differences in these features across age classes.

Unlike other primates, simakobu loud calls were not given to predators or during agonistic encounters or fights between adult males (e.g., chacma baboons [16], Thomas langurs [17], guenons, *Cercopithecus* spp. [59]). The fact that their calls do not serve as alarm calls could be related to the relative paucity of predators in the Mentawai Islands [49]. Although humans do frequently hunt simakobu, the monkeys typically respond to humans by fleeing or hiding, and were never observed to loud call in response to the presence of humans, even during habituation. Despite previous reports of males producing loud calls during intergroup encounters [43], [60], we never observed this behavior during the more than 50 encounters we witnessed. Furthermore, loud calls were not accompanied by the running or jumping displays exhibited by other species (e.g., Nilgir langurs, *Trachypithecus johnii* and Hanuman langurs, *Semnopithecus* sp. [61], purple-faced langurs, *Trachypithecus vetulus* [62], chacma baboons [8], ursine colobus monkeys, *Colobus vellerosus* [63]). These observations, together with the fact that calls typically elicit counter-calls from several hundred meters away, emphasize the role of these calls in long-distance extra-group communication.

Overall, loud call contexts did not show distinct acoustic differences. This result may indicate their universal function as advertisement signals, which convey information about the physical and physiological attributes of the caller rather than the external environment. The fact that simakobu loud calls are not used as alarm calls to predators suggests that the need for listeners to discriminate among various uncritical contexts may be less important. Furthermore, as contexts were defined by the auditory stimuli that preceded loud calls, it is possible that listeners also use this contextual information to distinguish the stimulus of the call [64]. Classification results should be interpreted with caution, however, as they may be sensitive to the number of contexts under consideration [65]. The differences that do exist among contexts could indicate the arousal or motivation of the caller. Spontaneous calls had higher peak frequencies than other calls, suggesting that the caller was more excited in these situations [31], [46]. Although we were unable to identify an auditory stimulus for these calls, it is possible that they may have been elicited by some distant visual or other sensory cue of which observers were unaware, as animals were often at heights exceeding 20 m.

In contrast, we found significant individual differences in simakobu loud calls. This result is not surprising, given that human observers could quickly differentiate callers, even at distances exceeding 200 m. Like many other studies, we found that the fundamental frequency contributed largely to the discrimination of individuals' calls (e.g., Thomas langurs [17], [41], Iberian wolves [24], fallow deer [29], red-capped mangabeys, *Cercocebus torquatus* [66]). We also found that temporal aspects of calls were important in distinguishing individual males. Although temporal features are often viewed as dynamic traits that vary with the arousal or motivation of the caller (cf. [30]), a number of studies have found distinct individual differences. Inter-unit intervals, for example, were among the most important features contributing to the discrimination of individual Thomas langurs' loud calls [41], while call duration was important for distinguishing adult male chacma baboons [16].

To test for short-term acoustic changes, which possibly reflect caller stamina and fatigue, we investigated changes in acoustic structure over the course of a single loud call. As loud calls progressed, we found that call units showed significant changes. We observed a significant decline in the fundamental frequency of the hoo syllables later in the call. This effect has also been documented in human speech and the vocalizations of vervet monkeys (*Cercopithecus aethiops*) and rhesus macaques (*Macaca mulatta*), and is due to the deflation of the lungs leading to a terminal decrease in the rate of vocal fold vibration [22]. In the vervets and macaques, this drop in fundamental frequency was also highly correlated with bout termination, and was hypothesized to serve a communicative role in vocal exchanges [67]. Thus, listening males might use this information to assess when a neighbor's call is nearing its end and decide when to begin their vocal responses.

We additionally found that the delivery of the calls slowed as the interval between successive call units increased over time. Call units also showed some higher frequency characteristics later in the calls, in particular the peak frequency and fundamental frequency of huh syllables. This result is similar to the pattern found for the loud “wahoo” calls given by male baboons, where wahoos produced early in calling bouts had a higher F0 than those later in the bouts [8]. While loud calls in male baboons are accompanied by lengthy aggressive displays, simakobu loud calls are shorter and do not involve this type of physical exertion. However, the loud calls of simakobu are delivered at a significantly faster rate (mean = 15.9 call units in 15.5 seconds) compared to those of baboons (mean = 20 wahoos in 120 seconds). In baboons, these vocal changes across the bout were interpreted as honest signals of caller exhaustion. Our findings are also in line with previous research that found the durations of simakobu loud calls were affected by short-term changes in the energy status of the caller [45]. Taken together, results suggest that loud calls may be honest and energetically costly signals of a male's competitive ability.

Possible long-term effects of the caller's strength and stamina were evaluated by examining the calls of males of different ages. We found that the fundamental frequency of the hoo was lowest and the inter-unit interval highest in the older age class. Similarly, in baboons, fundamental frequency decreased and call duration increased as adult males aged, even over periods as short as three years [8]. As these older males typically also dropped in rank over time, the changes in the fundamental frequency and duration of their calls appeared to honestly signal a reduction in fighting ability, rather than old age per se. A similar interpretation for simakobu may be supported by the fact that these same variables also showed short-term changes, over the course of a single loud call, perhaps indicating the caller's stamina and endurance.

Although we didn't make any predictions about changes in peak frequency with age, we did find that the peak frequency of the hoo syllable was highest in males of the middle age class. In humans and squirrel monkeys (*Saimiri sciureus*), peak frequency has been found to be the most important variable in the vocal expression of an aversive emotional state [31], [46]. In light of these studies, it is thus possible that this acoustic feature indicates a greater degree of arousal in males of the middle age class and perhaps a greater motivation to fight. Future research, ideally incorporating playback experiments, is needed to investigate this hypothesis.

Overall, the results of our study indicate that loud calls are individually distinct and may provide honest information about the caller. Listeners attending to the duration, inter-unit interval, fundamental frequency and peak frequency of calls could potentially assess the age, stamina, and arousal of the calling male, as well as short-term changes in energy status [45]. As in other species, these acoustic differences could thus be a way for males and females to discriminate neighbors from strangers (e.g., Thomas langurs [3]), identify previous rivals (e.g., feral horses, *Equus ferus* [68]), and assess competitors and mates from long distances (e.g., red deer [36]). Like other taxa, simakobu males likely use these loud calls to signal their presence and fighting ability in order to defend females and/or the resources within their home ranges. Playback experiments are needed to confirm this, by evaluating the salience of these features to listening males and females.
